# Function and Biosynthesis of Cell Wall α-1,3-Glucan in Fungi

**DOI:** 10.3390/jof3040063

**Published:** 2017-11-18

**Authors:** Akira Yoshimi, Ken Miyazawa, Keietsu Abe

**Affiliations:** 1 ABE-Project, New Industry Creation Hatchery Center, Tohoku University, 6-6-10 Aoba, Aramaki, Aoba-ku, Sendai, Miyagi 980-8579, Japan; a-yoshimi@niche.tohoku.ac.jp; 2 Laboratory of Applied Microbiology, Department of Microbial Biotechnology, Graduate School of Agricultural Sciences, Tohoku University, 468-1 Aoba, Aramaki, Aoba-ku, Sendai, Miyagi 980-0845, Japan; ken.m.tubbt@gmail.com; 3 Department of Microbial Resources, Graduate School of Agricultural Science, Tohoku University, 468-1 Aoba, Aramaki, Aoba-ku, Sendai, Miyagi 980-0845, Japan

**Keywords:** α-1,3-glucan, cell wall, filamentous fungi, dimorphic yeast, virulence factor, aggregation factor, *Aspergillus*

## Abstract

Although α-1,3-glucan is a major cell wall polysaccharide in filamentous fungi, its biological functions remain unclear, except that it acts as a virulence factor in animal and plant pathogenic fungi: it conceals cell wall β-glucan on the fungal cell surface to circumvent recognition by hosts. However, cell wall α-1,3-glucan is also present in many of non-pathogenic fungi. Recently, the universal function of α-1,3-glucan as an aggregation factor has been demonstrated. Applications of fungi with modified cell wall α-1,3-glucan in the fermentation industry and of in vitro enzymatically-synthesized α-1,3-glucan in bio-plastics have been developed. This review focuses on the recent progress in our understanding of the biological functions and biosynthetic mechanism of cell wall α-1,3-glucan in fungi. We briefly consider the history of studies on α-1,3-glucan, overview its biological functions and biosynthesis, and finally consider the industrial applications of fungi deficient in α-1,3-glucan.

## 1. Introduction

The fungal cell wall, which has a complex and dynamic structure and is mainly composed of polysaccharides, plays an essential role in defining cell shape and shielding the cells from environmental stresses, including changes in osmolality, temperature, and pH. These stresses primarily affect fungal cell walls and necessitate cell wall remodeling or repair. In animal and plant pathogenic fungi, cell walls are the first fungal elements to interact with host cells. Understanding of cell wall architecture is important for the control of fungal pathogens and also for the fermentation industry, including the production not only of fermented foods, such as sake (rice wine), shoyu (soy sauce), and miso (soybean paste), but also of various enzymes (e.g., amylases, lipases, and proteases) and chemicals (e.g., drug derivatives). Proper cell wall architecture in filamentous fungi requires several polysaccharides, including α-glucans (mainly α-1,3-glucan with low proportions of α-1,4-glucan), β-glucans (β-1,6-branched β-1,3-glucan), galactomannan, and chitin [1,2]. In addition, cell surfaces of some filamentous fungi are covered with secreted compounds, collectively called the extracellular matrix (ECM), which are mainly composed of polysaccharides [3,4]. Proteins, such as galactomannoproteins and GPI-anchored and surface proteins, are also present in cell walls of some filamentous fungi [1,5]. Although α-1,3-glucan is absent in the cell wall of the budding yeast *Saccharomyces cerevisiae*, it is a major component in the cell wall of filamentous fungi and dimorphic yeasts. The existence and importance of cell wall α-1,3-glucan were recognized well before the whole-genome analysis of some fungi [6,7]. Since α-1,3-glucan is accumulated in the cell wall in the early stage of *Aspergillus nidulans* culture and is degraded under carbon-limited conditions [8], it was thought to act as a storage polysaccharide in some fungi [8]. However, little was known about other biological functions of fungal α-1,3-glucan until whole genomes of fungi were sequenced. Whole-genome analyses of *Aspergillus* species performed since 2005 enabled the study of various functions of α-1,3-glucans in these species.

In the pathogenic dimorphic yeast *Histoplasma capsulatum* and the pathogenic filamentous fungus *Aspergillus fumigatus*, the cell wall polysaccharides, such as β-1,3-glucan and chitin, are recognized by host cells as pathogen-associated molecular patterns (PAMPs), and the fungal cells are attacked and removed by host immune systems [9,10,11]. In these fungi, cell wall α-1,3-glucan contributes to pathogenesis by concealing the PAMPs from detection by the host immune system [9,10,11]. In the rice blast fungus *Magnaporthe grisea*, cell wall α-1,3-glucan also acts as a stealth factor by blocking host recognition of fungal invasion and is required for infection of live rice cells [12,13]. Why do non-pathogenic fungi also possess α-1,3-glucan as a major cell wall component? In these fungi, α-1,3-glucan appears to function as an aggregation factor for hyphae and conidia, because the defect in α-1,3-glucan in some *Aspergillus* species leads to hyphal and conidial dispersion in medium [14,15,16]. The latter property of α-1,3-glucan has been used to improve fermentation productivity [17]. There has also been progress in application of α-1,3-glucan as a bio-based polymer [18]. In this review, we focus on recent progress in our understanding of the biosynthetic mechanism and biological functions of α-1,3-glucan in fungi.

## 2. Brief History of Studies on α-1,3-Glucan in Fungi

### 2.1. Functional Analysis of α-1,3-Glucan in Aspergillus nidulans before Whole-Genome Sequencing

In the early stages of research on biological functions of cell wall α-1,3-glucan in filamentous fungi, Zonneveld performed biochemical analysis in the model fungus *Aspergillus nidulans* [7,8,19,20,21,22]. First, he demonstrated that the main components of the mycelial cell wall in *A. nidulans* were glucose and acetylglucosamine, with minor quantities of mannose, galactose, galactosamine, protein, and lipid [7]. Zonneveld also showed that cell wall polysaccharides could be separated on the basis of alkali-solubility: one polymer was an alkali-soluble glucan and contained only α-1,3-glycosidic linkages, and the other component was alkali-resistant and contained chitin (about 50%) and α-1,4- and β-1,3-linked glucans [7].

He also identified a new type of enzyme, an exo-splitting α-1,3-glucanase, in six-day cultures of *A. nidulans* [8]. He found that the production of α-1,3-glucanase secreted by *A. nidulans* rapidly increased when glucose in the culture medium was depleted and some amount of its substrate, α-1,3-glucan, was present in the cell wall [19]. Under these conditions, α-1,3-glucan served as a carbon source, and cleistothecium formation started [19].

Zonneveld also reported the cellular responses of *A. nidulans* to 2-deoxy-d-glucose (2-DG), which is an analog of glucose and inhibits the growth and metabolism of many cell types [20]. When 2-DG was added to a culture of *A. nidulans* at the time of inoculation, biosynthesis of α-1,3-glucan, production of α-1,3-glucanase, and cleistothecium formation were inhibited. When 2-DG was added after cell wall α-1,3-glucan had been partly synthesized, production levels of α-1,3-glucanase were reduced, cleistothecium formation was inhibited, and the amount of the alkali-soluble fraction did not decrease. Zonneveld predicted that 2-DG would primarily inhibit the synthesis of α-1,3-glucan and resulted in the reduction of α-1,3-glucanase, whereas the decrease in α-1,3-glucanase production was attributable to a secondary effect of 2-DG. He also suggested that both α-1,3-glucan and α-1,3-glucanase were indispensable for fructification in *A. nidulans* [20].

Using nine *A. nidulans* mutants differing in the development of cleistothecia and/or conidia, Zonneveld investigated the relationships between these phenotypes and α-1,3-glucan or α-1,3-glucanase [21]. Low levels of cell wall α-1,3-glucan in mycelia on the third day of plate culture were associated with low α-1,3-glucanase activity in the medium and the absence of cleistothecium formation on the sixth day. The level of α-1,3-glucan and cleistothecium formation seemed to be inversely related to conidiation. Zonneveld also analyzed the influence of manganese in media on cleistothecium development in *A. nidulans*, because manganese was thought to be required for sporulation and protease production in some bacteria and fungi. Manganese deficiency prevented cleistothecium development, but vegetative growth was not altered. α-1,3-Glucan was absent from the mycelial cell wall under manganese deficiency [22].

Polacheck and Rosenberger [23] isolated a melanin-deficient mutant of *A. nidulans* and found that the mutant lost most of the cell wall α-1,3-glucan, formed no cleistothecia, and contained increased levels of β-1,3-glucan and galactose polymers in the cell wall; electron micrographs indicated that mutant cells had lost the outermost wall layer. The cell wall of the mutant was more readily digested by lytic enzymes than that of the wild type, and this increased susceptibility to hydrolysis was caused by the absence of α-1,3-glucan, but not by that of melanin. These results were consistent with α-1,3-glucan acting as an endogenous carbon source for biosynthetic processes in the stationary phase of growth [23].

A subtractive cDNA library of *A. nidulans* was established to identify genes differentially expressed during sexual development, and a clone with sequence similarity to fungal α-1,3-glucanases (mutanases) was identified [24]. Transcript analysis of the corresponding gene, named *mutA*, and *mutA* promoter–GFP reporter analysis in *A. nidulans* revealed specific induction of the gene during sexual development in Hülle cells. The number of cleistothecia formed by a deletion strain lacking *mutA* was similar to that of the wild-type [24]. Unlike previous reports [20,22,23], these results suggested that α-1,3-glucan is dispensable for sexual development because some additional carbon sources might be available.

### 2.2. Functional Analysis of α-1,3-Glucan in Aspergillus Species after Complete Genome Sequences Became Available

The whole genomes of several *Aspergillus* species have been sequenced [25,26,27,28], which greatly accelerated the analyses of α-1,3-glucan synthase (*AGS*) genes. At least two *AGS* genes have been identified in *A. nidulans* (*agsA* and *agsB*), *A. oryzae* (*agsA*, *agsB*, and *agsC*), *A. fumigatus* (*AGS1*, *AGS2*, and *AGS3*), and *A. niger* (*agsA*, *agsB*, *agsC*, *agsD*, and *agsE*) [2] (Table 1).

*Aspergillus fumigatus AGS1* and *AGS2* are orthologous to *A. nidulans agsB* and *agsA*, respectively. Disruption of either *AGS1* or *AGS2* in *A. fumigatus* reduced conidiation and caused abnormal hyphal apices and abnormal phialide formation. Disruption of *AGS1* but not *AGS2* decreased cell wall α-1,3-glucan content by 50% [29]. Deletion of *A. fumigatus AGS3*, which has no *A. nidulans* ortholog, did not change cell wall composition increased *AGS1* expression, likely to compensate for *AGS3* deficiency [30]. *AGS3* disruption led to a three-fold increase in melanin content in the conidia, improved resistance to reactive oxygen species, and accelerated germination [30]. A triple *AGS* disruptant (Δ*ags*) strain lost cell wall α-1,3-glucan, and its germinating conidia did not aggregate [15]. Whereas the wild-type conidia were covered with a crystalline-like array of rodlets, the rodlet layer on the Δ*ags* mutant conidial surface was covered by an amorphous glycoprotein matrix [11].

In *A. niger*, the expression of *agsA* (orthologous to *A. fumigatus AGS3*) and *agsE* (orthologous to *A. fumigatus AGS1*) was induced in the presence of the cell wall stress-inducing compounds calcofluor white, sodium dodecyl sulfate, and caspofungin, an inhibitor of β-1,3-glucan synthase [31]. 

Fujioka et al. analyzed target genes downstream of the cell wall integrity signaling (CWIS) cascade in *A. nidulans* [32]. In the wild-type strain, treatment with a β-1,3-glucan synthase inhibitor (micafungin) activated the MAP kinase MpkA, as in the case of its ortholog Mpk1p (Slt2p) in *S. cerevisiae* [32]. An *A. nidulans mpkA* disruptant was used to investigate the cell wall stress response to micafungin by analyzing cell wall synthase. The transcription of the β-1,3-glucan synthase gene *fksA* and chitin synthase genes (*chsA–D*, *csmA*, and *csmB*) was regulated independently of the MpkA pathway, whereas the transcription α-1,3-glucan synthase genes (*agsA* and *agsB*) depended on MpkA. In the *mpkA* disruptant, *agsA* expression was slightly upregulated, and that of *agsB* was markedly downregulated. Although the yeast model had been applied to the CWIS pathway in filamentous fungi, it has been suggested that the role of the orthologous pathway in cell wall synthesis is quite different in these fungi [32].

In *A. nidulans*, the expression level of *agsA* is extremely low, but that of *agsB* was rather high under normal growth conditions. The cell wall of the ∆*agsB* and ∆*agsA*∆*agsB* strains lacked α-1,3-glucan, suggesting that AgsB plays a major role in α-1,3-glucan synthesis [16]. Hyphae of the ∆*agsB* and ∆*agsA*∆*agsB* strains dispersed evenly in liquid medium, indicating that the cell wall α-1,3-glucan functions as a hyphal aggregation factor in *A. nidulans* [16]. In addition, the ∆*agsB* and ∆*agsA*∆*agsB* strains showed increased sensitivity to Congo red (CR), which interacts with β-1,3-glucan and chitin [16]. Significantly less CR was adsorbed to α-1,3-glucan than to β-1,3-glucan or chitin [16]. These results suggest that the loss of cell wall α-1,3-glucan in the ∆*agsB* and ∆*agsA*∆*agsB* strains increased the exposure of β-1,3-glucan on the cell surface and therefore increased sensitivity to CR [16].

He et al. [33] reported the function of two α-glucan synthases (AgsA and AgsB) and two α-amylases (AmyD and AmyG) in *A. nidulans.* AmyD, but not AmyG, has a signal peptide and a GPI-anchor site. The *amyD* and *amyG* genes form a cluster with the *agsB* gene [33]. In liquid cultures, an *amyG* disruptant and an *agsB* disruptant formed similar small pellets of hyphae and showed a significant loss of cell wall α-1,3-glucan. Consistent with the absence of a signal peptide, AmyG-GFP tagging revealed that AmyG is a cytoplasmic protein. *Histoplasma capsulatum* AMY1 and *A. niger* AmyD, both of which are orthologous to *A. nidulans* AmyG, are thought to be involved in synthesizing the primer (α-1,4-linked glucooligosaccharide), which is linked to the reducing end of α-1,3-glucan and stabilizes it in vivo [33]. Thus, it is likely that AmyG synthesizes the primer structure for the synthesis of α-1,3-glucan [33]. On the other hand, the *amyD* disruptant had more cell wall α-1,3-glucan than the wild-type strain [33]. AgtA of *A. niger*, an ortholog of AmyD of *A. nidulans*, is a glucanotransferase that acts on α-1,4-glycosidic bonds [34]. Based on these findings, He et al. [33] hypothesized that AmyD might degrade the oligo-α-1,4-glucan primer, indirectly weakening α-1,3-glucan protection from degradation by α-1,3-glucanase. However, there is some room for debate on this hypothesis [33,34].

Since α-1,3-glucan was considered to be important in the sexual development of *A. nidulans* [21,23], He et al. [33] performed a mating experiment between the α-1,3-glucan-deficient and wild-type strains, but found no difference in mating in respect to the formation of cleistothecia and the number of ascospores in each cleistothecium. Similarly, we found no difference in cleistothecium and ascospore formation between our *A. nidulans* α-1,3-glucan-deficient strains and the wild-type strain [35]. Thus, it is likely that α-1,3-glucan does not affect sexual development in *A. nidulans*.

## 3. Biosynthesis of α-1,3-Glucan

### 3.1. Regulation of α-1,3-Glucan Biosynthesis in Filamentous Fungi: Cell Wall Integrity Signaling

**Figure 1:**
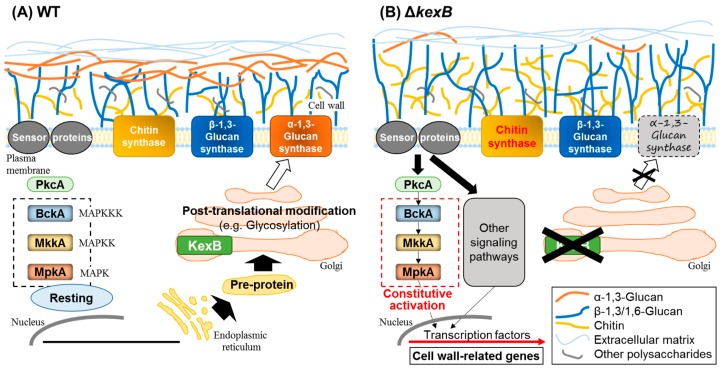
Maintenance of cell wall integrity by KexB in *Aspergillus oryzae*. KexB is predicted to be required for correct proteolytic processing of sensor proteins and cell wall–related enzymes, including α-1,3-glucan synthase. (**A**) In the wild-type (WT) strain, the cell wall integrity MAP kinase pathway (dashed square) is not activated (resting) under normal growth conditions (no cell wall perturbation). Pre-protein is transported from the endoplasmic reticulum to the Golgi apparatus, and is subsequently modified (e.g., glycosylated). (**B**) In the Δ*kexB* strain, the cell wall integrity pathway is constitutively activated, resulting in a substantial decrease in cell wall α-1,3-glucan. This decrease upregulates the transcription of cell wall–related genes and is compensated by an increase in chitin content and an increase in the degree of β-1,3-glucan polymerization. The crosses indicate no-function.

For many years, the yeast model was applied to the CWIS pathway in *Aspergillus* species. The CWIS pathway in *S. cerevisiae* consists of sensor proteins on the cell surface, protein kinase C, and the downstream MAP kinase pathway [40]. Cell wall perturbations are sensed by the sensor proteins, such as Wsc1p, Wsc2p, and Wsc3p, which are highly glycosylated [41,42]. The cell wall perturbation signal is first transmitted to the small G protein Rho1p [43], which is activated by the guanosine nucleotide exchange factors (GEFs) Rom1p, Rom2p, and Tus1p [44,45]. Active Rho1p binds to, and activates, protein kinase C (Pkc1p) which, in turn, activates the mitogen-activated protein kinase (MAPK) cascade [46]. The MAPK cascade is composed of the MAPK kinase kinase Bck1p [47], a pair of redundant MAPK kinases, Mkk1p and Mkk2p [48], and the MAPK Mpk1p/Slt2p [49]. As shown in Figure 1, an orthologous MAPK cascade is conserved in *Aspergillus*. Phosphorylated Mpk1p activates the transcription factor Rlm1p, which regulates transcription of at least 25 genes involved in cell wall biogenesis, including the β-1,3-glucan synthase and chitin synthase genes [50].

Whole genome sequencing revealed that the homologous CWIS pathway and cell wall–related genes are conserved in *A. nidulans*, *A. oryzae*, and *A. fumigatus* [32]. In an *A. nidulans* wild-type strain, almost all genes involved in cell-wall biogenesis were upregulated transiently by micafungin. In the deletion strains lacking *mpkA* or *rlmA*, which are orthologous to *S. cerevisiae mpk1* and *rlm1*, respectively, the genes for β-1,3-glucan and chitin synthases were upregulated by micafungin similarly to the wild-type. On the contrary, the transcription of *agsB* was significantly downregulated in the *mpkA*Δ and *rlmA*Δ strains regardless of micafungin treatment. The *agsA* gene was transcribed at a very low level in wild-type *A. nidulans* strains, but its transcription was weakly upregulated in the *mpkA*Δ and *rlmA*Δ strains, but not in the wild-type strain when the cells were treated with micafungin. Therefore, the CWIS pathway in *A. nidulans* is regulated somewhat differently from that in *S. cerevisiae*; in particular, the *A. nidulans* MpkA pathway regulates mainly cell wall α-1,3-glucan biogenesis [32].

In *A. oryzae*, the CWIS pathway has been studied by analyzing the functions of the subtilisin-like processing protease KexB, a Kexin-like enzyme orthologous to *S. cerevisiae* Kex2p [51,52]. Kexins are Ca^2+^-dependent transmembrane serine proteases that cleave secretory pro-proteins on the carboxyl side of Lys-Arg and Arg-Arg in the late Golgi compartment [53,54]. An *A. oryzae* strain with a deletion of the *kexB* gene (∆*kexB*) formed shrunken colonies with poor generation of conidia on Czapek–Dox (a minimal medium for *Aspergillus* species) agar plates and hyperbranched mycelia in Czapek–Dox liquid medium. In ∆*kexB*, the transcript levels of the *mpkA* gene and cell wall–related genes, such as β-1,3-glucanosyltransferase and chitin synthases, were significantly higher than in the wild-type strain, and MpkA was constitutively phosphorylated. The upregulation of the transcription is attributable to the autoregulation by phosphorylated MpkA as the active form through cell integrity signaling (Figure 1). High osmotic stress restored the growth defects in the ∆*kexB* strain and downregulated the increased levels of both transcripts of *mpkA* and the phosphorylated form of MpkA [51].

Mizutani et al. [52] hydrolyzed the total cell wall fraction and analyzed carbohydrate composition to determine the effect of *kexB* deletion on cell wall components. In the ∆*kexB* strain, the content of chitin (determined as the content of *N*-acetylglucosamine; GlcNAc) was about 1.5 that in the wild type. This increase was consistent with the constitutive transcriptional activation of chitin biosynthetic genes in ∆*kexB*. Total content of β-1,3- and α-1,3-glucans (determined as glucose content) was decreased; further analysis indicated a remarkable decrease in α-1,3-glucan. Since the predicted amino acid sequences of the α-1,3-glucan synthases (AgsA, AgsB, and AgsC) in *A. oryzae* share a KexB recognition sequence in the N-termini, KexB might be involved in their processing. Thus, the Ags may not function in the Δ*kexB* strain. The degree of polymerization of β-1,3-glucan derived from the Δ*kexB* strain was 1.5 that in the wild-type strain. Therefore, the loss of α-1,3-glucan was compensated by an increase in chitin content and in the degree of β-1,3-glucan polymerization (Figure 1). The above data indicate that *A. oryzae* KexB is indispensable for the biosynthesis of the cell wall and the maintenance of its integrity. However, it is unclear why the degree of β-1,3-glucan polymerization is increased in the Δ*kexB* strain, and whether the overexpression of cell wall–related genes in Δ*kexB* depends on MpkA.

### 3.2. Biosynthesis of α-1,3-Glucan in Schizosaccharomyces pombe

Hochstenbach et al. [55] and Vos et al. [56] predicted the structure and function of the *Schizosaccharomyces pombe* α-1,3-glucan synthase Ags1p, which contains two probable catalytic domains for α-glucan assembly. It contains an extracellular domain, a transmembrane region, an intracellular domain, and a multitransmembrane domain. The N-terminus of Ags1p contains a cleavable signal peptide [55]. The putative intracellular domain contains an N-terminal Lys-rich region and a C-terminal Ser-rich region; the region between them (SYN domain) shares sequence similarity with glycogen and starch synthases [56]. Vos et al. [56] created an *S. pombe* strain overexpressing Ags1p-E1526A, which had a mutation of a predicted catalytic residue in the SYN domain. Staining of *S. pombe* cells overexpressing Ags1p with iodine vapor, which stains α-1,4-glucan (see the description of the SYN domain below), suggested the accumulation of α-1,4-glucan, whereas no α-1,4-glucan accumulation was observed in the strain overexpressing Ags1p-E1526A [56]. Ags1p lacking the SYN domain also did not induce accumulation of α-1,4-glucan. These results suggest that the SYN domain is responsible for the production of α-1,4-glucan.

The putative extracellular domain of Ags1p shows high sequence similarity to bacterial α-amylases of the glycosyl hydrolase family 13 (GH13) [55]. The cells of the temperature-sensitive mutant strain *ags1-1*^ts^, which carries an amino acid substitution in the extracellular domain of Ags1p, were rounded or pear-shaped when grown at 34 °C, and their levels of cell wall α-glucan were about 1/3 that of the wild-type [55]. At 37 °C, cell lysis was observed after 5 h, and most cells were lysed by 12 h. In *ags1-1*^ts^ cells grown at 34 °C, cell wall α-glucan was composed of a single chain of about 120 1,3-linked α-glucose residues (about half of the length in the wild-type) and some 1,4-linked α-glucose residues at the reducing end [57]. Therefore, the extracellular domain of Ags1p was suggested to transfer an α-glucose residue to other α-glucose residues [57].

Grün et al. [57] analyzed the chemical structure of cell wall α-glucan of fission yeast to gain insight into the molecular mechanism of its biosynthesis. Size-exclusion chromatography revealed a number-average molecular mass (M_n_) of 42.6 ± 5.2 kDa and a number-average degree of polymerization (DP_n_) of 263 ± 32 in wild-type cells. α-Glucan showed a polydispersity of 2.41 ± 0.25 (weight-average molecular mass (M_w_)/M_n_), indicating a relatively narrow molecular mass distribution. Linkage analysis by methylation showed that α-glucan consisted of 1,3-linked glucose residues (88.9 ± 1.0%) and 1,4-linked residues (9.0 ± 0.3%), and had no branching points. Heteronuclear single-quantum correlation NMR analysis revealed less than 9% of 1,4-linked α-glucose residues. The results of Smith degradation suggested that 1,4-linked glucose residues are located in the center and interconnect two chains of ~120 1,3-linked glucose residues, whereas β-elimination analysis demonstrated the presence of 1,4-linked α-glucose residues at the reducing end. The extracellular transglycosylase domain of Ags1p links together two ~120 chains of α-glucan [57]. The SYN domain may be involved in elongation of α-1,3-glucan homopolymers at the plasma membrane. They must be transported outside the cell via a pore-like structure, which might be formed by the multitransmembrane domain of Ags1p [55].

The biosynthesis of α-1,4-glucan requires initiation and elongation steps. In mammals and yeasts, glycogen synthesis is initiated by autoglucosylation of the protein glycogenin to form glycogenin-bound Tyr-194-linked α-1,4-oligoglucan, which may act as a primer for elongation by glycogen synthase [58]. The SYN domain of *S. pombe* Ags1p might be responsible for elongation of α-1,4-glucan [56]. However, the *S. pombe* genome does not encode a readily identifiable glycogenin-like protein, and further studies are required to identify the initiation factor in α-1,4-glucan biosynthesis. In dimorphic yeasts such as *H. capsulatum* and in filamentous fungi, such as *A. nidulans*, intracellular amylases might synthesize the primer for α-1,3-glucan biosynthesis [33,38,59,60]. The α-1,3-glucan synthases of these fungi possess the three conserved domains found in *S. pombe* Ags1p. However, a defect in intracellular amylase leads to a significant decrease in cell wall α-1,3-glucan content, suggesting that the mechanisms of α-1,3-glucan biosynthesis in dimorphic yeasts and filamentous fungi are quite different from that in the fission yeast. Further studies, including the search for initiation factors and the development of an in vitro assay for α-1,3-glucan synthase activity, are needed for elucidation of detailed mechanisms of α-1,3-glucan biosynthesis in fungi.

In *Aspergillus wentii*, the alkali-soluble fraction of the cell wall is composed mainly (>99%) of α-1,3-linked glucan that contains a small amount of probable spacer α-1,4-linked glucan incorporated into each α-1,3-glucan unit [61]. Smith degradation revealed that *A. wentii* cell wall α-1,3-glucan consists of approximately 25 subunits, each built of about 200 1,3-linked α-glucose residues separated by several short spacers of α-1,4-linked glucosyl oligosaccharide [61].

Structural characterization of α-1,3-glucan was reported in *A. fumigatus*, *A. nidulans*, *A. niger*, and *A. wentii* [62]. Linkage analysis by methylation revealed that alkali-soluble polysaccharides were polymers composed of 91–98% of 1,3-linked glucose, 1–8% of 1,4-linked glucose, and traces of 1,3/1,6 branched residues [62]. Fourier transform infrared spectroscopy analysis confirmed that alkali-soluble polysaccharides from each fungus are mainly composed of α-1,3-glucan.

### 3.3. Intracellular Amylase

In some fungi, including *H. capsulatum*, α-1,3-glucan production requires not only α-1,3-glucan synthase but also intracellular α-amylase. α-Amylase is involved in the synthesis of an oligosaccharide of (1,4)-linked α-d-glucose residues, which serves as a primer for α-1,3-glucan chain initiation at the non-reducing end of the primer [59]. Intracellular α-amylases in *Aspergillus* species and pathogenic yeasts are classified into the glycoside hydrolase subfamily GH13_5 [38,60]. These α-amylases have four highly-conserved regions, and shared common features with most eukaryotic intracellular α-amylases and bacterial α-amylases of the liquefying type (GH13_5; http://www.cazy.org/GH13_5.html) [36,60]. These features include: (i) a histidine (H52) and a cysteine (C79); in the regions flanking the β2 strand at its N-terminus and C-terminus, respectively; (ii) an invariant leucine residue before the conserved NH motif in conserved region I, at the end of the β3 strand; (iii) two aromatic residues following the catalytic glutamate proton donor in conserved region III (around strand β5); and (iv) cysteine and leucine residues in the β8 strand.

The intracellular α-amylase of *A. niger*, AmyD, has a relatively low activity on starch and amylopectin, initially producing mainly maltotriose with subsequent production of maltose and several larger maltooligosaccharides, mainly with a DP of 6–9 [38]. Neither dextran, α-1,3-glucan, nigeran, nor *Lactobacillus reuteri* exopolysaccharide (containing α-1,3 and α-1,6-glucosidic bonds) is hydrolyzed by AmyD [38]. AmyD has low activity towards amylopectin and almost no activity towards glycogen. When AmyD was incubated with trehalose, sucrose, UDP-glucose, nigerose, or nigerotriose as sole substrates or in combination with starch or maltoheptaose, no reaction products other than those resulting from the hydrolysis of starch or maltoheptaose were detected [34].

The intracellular α-amylase of *P. brasiliensis* Amy1p showed a higher activity on amylopectin than on starch, producing oligosaccharides of 4 or 5 glucose residues [60].

In *A. nidulans*, the intracellular α-amylase gene, *amyG*, forms a cluster with the genes for the GPI-anchored amylase (*amyD*) and the α-1,3-glucan synthase (*agsB*) [33]. The *amyG* transcript level is not upregulated by carbon starvation [63]. The deletion of *amyG* decreased cell wall α-1,3-glucan content, so it is likely that AmyG synthesizes the primer structure for the synthesis of α-1,3-glucan [33]. 

A gene cluster composed of one α-1,3-glucan synthase gene and two α-amylase genes is conserved in *Aspergillus* species [2,33]. The cluster components are *agsE*, *agtA*, and *amyE* in *A. niger* and *agsB*, *amyD*, and *amyG* in *A. oryzae*. In *A. fumigatus*, *AMY1* (ortholog of *A. nidulans amyD*) forms a cluster with the α-1,3-glucan synthase gene *AGS1*, but the intracellular α-amylase gene (AFU1G_15150) is located on another chromosome. There are variations in the number of intracellular α-amylase genes among *Aspergillus* species: one in *A. nidulans* and *A. fumigatus*, and two in *A. niger* and *A. oryzae*.

Taken together, the available data suggest that the α-1,4-oligosaccaride synthesized by intracellular α-amylase is crucial for α-1,3-glucan production in filamentous fungi and dimorphic yeasts. Further research on intracellular α-amylases will be required to clarify the details of the enzymatic reaction they catalyze, including the identification of their substrate.

### 3.4. Other Enzymes Involved in α-1,3-Glucan Synthesis

Unlike AmyG, AmyD of *A. nidulans* has a signal peptide and a GPI-anchor site, and is a membrane-associated protein [33,37]. Carbon starvation did not affect the transcript levels of *amyD* [63] Treatment with calcofluor white increased the *amyD* transcript level [33]. The transcript level was markedly higher at 24 h than at 14 h in the absence of calcofluor white in both shaken and static liquid cultures [33]. The amount of cell wall α-1,3-glucan was higher in the ∆*amyD* strain than in the wild-type strain, but was decreased by the overexpression of *amyD* [33,37]. From these results, He et al. [37] predicted that AmyD directly suppresses α-1,3-glucan synthesis in *A. nidulans*.

In *A. niger*, the function of the GPI-anchored amylase, AgtA (ortholog of *A. nidulans* AmyD), and AgtB was analyzed [34]. AgtA-SBD, which lacked its C-terminal anchoring domain and was fused to the starch-binding domain (SBD) of *A. niger* glucoamylase, hydrolyzed starch and produced glucose, maltose, or longer maltooligosaccharides. When AgtA-SBD was incubated with maltopentaose or maltoheptaose, a variety of oligosaccharides ranging from maltose to oligosaccharides with a DP of at least 13 to 18, respectively, were formed. Both AgtA and AgtB had a low level of starch hydrolyzing activity and did not hydrolyze dextran, nigeran, an *L. reuteri* polysaccharide containing α-1,3- and α-1,6-glycosidic bonds, or cellulose (β-1,4-glycosidic bonds). Both AgtA and AgtB produced a variety of oligosaccharides with a DP of up to 28 with maltopentaose or maltohexaose as substrates. When AgtA was incubated with soluble starch as a donor substrate in the presence of glucose, maltose, nigerose, or nigerotriose, it used maltose, nigerose, and nigerotriose as acceptor substrates. In contrast, AgtB did not use nigerose or nigerotriose efficiently. These results suggest that small α-1,3-linked oligosaccharides can be used as acceptor substrates by AgtA, but only in combination with an α-1,4-linked donor molecule.

Recently, He et al. [37] reported the function of AmyD, AgnB, and MutA in *A. nidulans*. Since AgtA of *A. niger* does not hydrolyze α-1,3-glucan and has low starch hydrolysis ability, it was thought that AmyD of *A. nidulans* hydrolyzes α-1,4-glucan linked to the end of α-1,3-glucan, thus accelerating α-1,3-glucan degradation by α-1,3-glucanase [33]. The overexpression of *mutA* and *agnB*, both of which encode α-glucanases, with or without *amyD* deletion similarly reduced α-1,3-glucan content. Therefore, the functions of glucanases are independent of AmyD. At 16 h of culture, the cell wall α-1,3-glucan content was almost the same in *agnB-* and *mutA*-overexpressing strains and in the wild type, but it was only half that level in an *amyD*-overexpressing strain. After 24 h, the content of cell wall α-1,3-glucan was only about half the wild-type level in all three overexpressing strains. He et al. [37] predicted that AmyD, AgnB, and MutA decrease cell wall α-1,3-glucan content through different mechanisms, although the mechanisms remain unclear. AmyC and AmyE share a considerable sequence identity with AmyD; however, the overexpression of *amyC* and *amyE* did not affect α-1,3-glucan synthesis in *A. nidulans* [37]. Nitsche et al. [64] identified some carbon starvation–induced glycoside hydrolases by transcriptome and secretome analysis in *A. niger*. In the same species, van Munster et al. reported that the transcription of the α-1,3-glucanase gene *agnB* was notably increased during carbon starvation [39]. AgnB liberated glucose from α-1,3-glucan. A double disruptant of *agnB* and the chitinase gene *cfcA* had reduced cell wall degradation during carbon starvation. These results suggest that AgnB hydrolyses cell wall during carbon starvation [39].

In *H. capsulatum*, the repression of the *UGP1* gene, which encodes UTP-glucose-1-phosphate uridylyltransferase, by RNA interference (RNAi) decreases the content of cell wall α-1,3-glucan, suggesting that UGP1 might function in α-1,3-glucan production in this dimorphic yeast [59].

**Figure 2:**
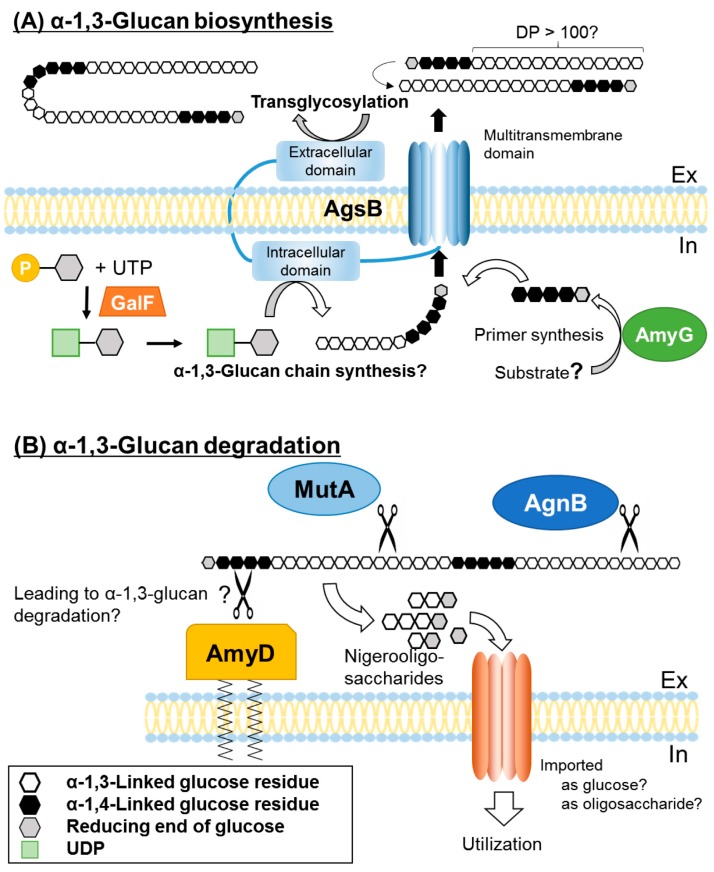
Speculative model for biosynthesis and degradation of α-1,3-glucan in *Aspergillus nidulans*. (**A**) Cell wall α-1,3-glucan is mainly synthesized by AgsB. The putative amino acid sequence of AgsB is similar to that of Ags1p (*Schizosaccharomyces pombe* α-1,3-glucan synthase). Both proteins contain an extracellular domain, an intracellular domain, and a multitransmembrane domain. The substrate for α-1,3-glucan biosynthesis, UDP-glucose, is synthesized by GalF, which is a homolog of *Histoplasma capsulatum* UGP1 (UTP-glucose-1-phosphate uridylyltransferase). α-1,4-Glucooligosaccharide is thought to be necessary as a primer for α-1,3-glucan biosynthesis and is predicted to be produced by the intracellular α-amylase AmyG. The α-1,3-glucan chain might be synthesized by the intracellular domain of AgsB and then exported outside of the cell through the multitransmembrane domain. In the extracellular domain, two exported chains of α-1,3-glucan, each connected to the non-reducing end of the α-1,4-oligoglucan primer, are combined by transglycosylation, resulting in mature α-1,3-glucan containing a small amount of α-1,4-glucooligosaccharide. DP, degree of polymerization. (**B**) Under carbon starvation conditions, cell-wall α-1,3-glucan serves as a carbon source. The GPI-anchored α-amylase AmyD hydrolyzes α-1,4-glucooligosaccharide linked to the end of cell wall α-1,3-glucan, which accelerates α-1,3-glucan degradation by α-1,3-glucanases such as MutA and AgnB. The detailed mechanisms of this degradation remain unclear. Nigerooligosaccharides and glucose derived from α-1,3-glucan degradation are predicted to be imported into the cell and used as carbon sources in fructification. In, intracellular; Ex, extracellular.

Based on these findings, we suggest the following model for α-1,3-glucan biosynthesis and degradation in *A. nidulans* (Figure 2). Cell wall α-1,3-glucan is synthesized by AgsB, which contains extracellular, intracellular, and multitransmembrane domains. The substrate for α-1,3-glucan biosynthesis, UDP-glucose, is synthesized by GalF, which is homologous to *H. capsulatum* UGP1. α-1,4-Glucooligosaccharide is likely necessary as a primer for α-1,3-glucan biosynthesis, and this oligosaccharide may be produced by the intracellular α-amylase AmyG. α-1,3-Glucan chain may be synthesized by the intracellular domain of AgsB, followed by its transport outside of the cell through the multitransmembrane domain. Then, two polysaccharide chains are connected by transglycosylation by the extracellular domain, resulting in mature α-1,3-glucan. Under carbon starvation conditions, cell wall α-1,3-glucan serves as a carbon source. The GPI-anchor-type α-amylase, AmyD, hydrolyzes α-1,4-glucooligosaccharide, which is linked to the reducing end of cell wall α-1,3-glucan, thus accelerating α-1,3-glucan degradation by the α-1,3-glucanases MutA and AgnB. Nigerooligosaccharide or glucose derived from α-1,3-glucan degradation is imported into the cells and is used as a carbon source.

## 4. Biological Functions of α-1,3-Glucan in Fungi

### 4.1. Function as an Aggregation Factor

**Figure 3:**
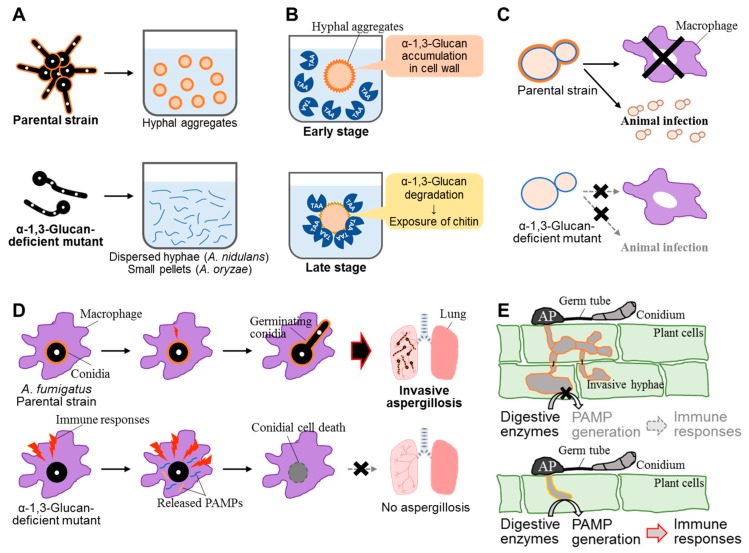
Various functions of α-1,3-glucan in fungi. (**A**) Swollen conidia of *Aspergillus* species aggregate in the presence of α-1,3-glucan, resulting in hyphal aggregates under liquid culture conditions. In contrast, germinating conidia of α-1,3-glucan-defcient mutants do not aggregate. The hyphae of these mutants are dispersed (*Aspergillus nidulans*) or form small pellets (*Aspergillus oryzae*) in liquid culture. (**B**) In the early stage of submerged culture of *A. oryzae*, TAA (Taka-amylase A) diffuses to liquid medium because α-1,3-glucan accumulates on the surface of the mycelial cell wall. In the late stage, cell wall α-1,3-glucan is degraded to be used as a carbon source, and chitin in the cell wall is exposed. Then TAA is adsorbed and retained on the cell surface. (**C**) In *Histoplasma capsulatum* chemotype II, α-1,3-glucan is found in the outer layer of the cell wall of the yeast form, where it covers β-1,3-glucan. The production of the cytokine TNFα by host macrophages is suppressed by α-1,3-glucan, resulting in the suppression of immune responses and pathogen’s colonization of host cells. The lack of α-1,3-glucan attenuates the ability of the pathogen to kill macrophages and to colonize lung cells. The crosses indicat no-invasion. (**D**) *Aspergillus fumigatus* conidia are rarely recognized by host macrophages because of the presence of α-1,3-glucan and a rodlet layer. Delayed host immune responses allow conidia to germinate. Conidia of the triple α-1,3-glucan synthase disruptant (Δ*ags*) are immediately recognized by macrophages and show an increased release of pathogen-associated molecular patterns (PAMPs), resulting in a strong innate immune response, The cross indicates no-infection. (**E**) In *Magnaporthe grisea*, α-1,3-glucan accumulates on the appressoria (AP) and germ tube, which makes them tolerant to chitinase digestion because β-1,3-glucan and chitin are masked by α-1,3-glucan. PAMP release is suppressed, and the pathogen infects the plant. In an α-1,3-glucan-deficient mutant, exposed β-1,3-glucan and chitin are digested by plant enzymes, resulting in release of PAMPs. The PAMPs are recognized by plant cells, leading to immune responses and pathogen killing. The cross indicates no-digestion.

In *A. fumigatus*, cell wall α-1,3-glucan is essential for aggregation between swollen conidia because their aggregation can be specifically prevented by the addition of α-1,3-glucanase (Figure 3A) [14]. The exposure of cell wall α-1,3-glucan chains on the cell surface during the swelling was demonstrated by electron microscopy. Experiments with α-1,3-glucan-coated latex beads showed inter-chain interaction that did not require any other cell wall component, suggesting that the biophysical properties of α-1,3-glucan are solely responsible for conidial aggregation [14]. As mentioned above, the hyphae of an *A. nidulans* α-1,3-glucan-deficient strain were completely dispersed in liquid medium (Figure 3A) [16]. Although the hyphae of an α-1,3-glucan-deficient strain in *A. oryzae* were not fully dispersed in liquid medium, the strain did form smaller hyphal pellets than the wild-type strain [17]. These observations suggest that the contribution of α-1,3-glucan as a hyphal or conidial aggregation factor might differ among fungal species. The avirulent strains of *H. capsulatum*, which form smooth-textured colonies on solid medium and have decreased cell wall α-1,3-glucan content, grow as dispersed cells in liquid medium [65]. Therefore, α-1,3-glucan might be involved in cellular aggregation and virulence in *H. capsulatum*.

### 4.2. Influence on Adsorption of α-Amylase onto the Cell Surface

Sato et al. [66] reported that the disappearance of α-amylase (Taka-amylase A; TAA) activity from the medium in the late stage of submerged *A. oryzae* culture was caused by the adsorption of TAA onto the cell wall, particularly chitin, not by TAA degradation by extracellular proteolytic enzymes (Figure 3B). Subsequently, Zhang et al. [36] analyzed the reason why TAA adsorption occurs only in the late stage and identified α-1,3-glucan as a potential factor inhibiting TAA adsorption. In the *A. oryzae* Δ*agsB* mutant, TAA was adsorbed onto the mycelia in all stages of submerged culture. In the wild-type strain, the α-1,3-glucan content of the cell wall was markedly lower in the late stage than in the early stage. These results suggest that α-1,3-glucan inhibits TAA adsorption onto another cell wall component, chitin, in the early stage of culture (Figure 3B) [36]. Taken together, the above data suggest the following: When nutrients are sufficient in liquid culture and fungal cells are growing, α-1,3-glucan is produced and incorporated into the cell wall. When nutrients are depleted, cell wall α-1,3-glucan is degraded and used as a carbon source. Along with α-1,3-glucan degradation, cell wall chitin becomes exposed, and TAA is adsorbed and retained on the cell surface. As a result, TAA can immediately react with its substrate (e.g., starch or maltooligosaccharide) efficiently on the cell surface in the poor-nutrient environment when *A. oryzae* cells encounter such substrates. The reaction products such as glucose and maltooligosaccharides can be used efficiently by hyphal cells.

### 4.3. Function as a Virulence Factor

#### 4.3.1. Relationships between α-1,3-Glucan and Virulence in Pathogenic Yeasts

In pathogenic dimorphic yeasts, such as *H. capsulatum*, *Paracoccidioides brasiliensis*, and *Blastomyces dermatitidis*, cell-wall α-1,3-glucan is related to their virulence (Figure 3C) [65,67,68,69,70,71,72]. San-Blas et al. [67] analyzed the phenotype of the *P. brasiliensis* mutant IVIC Pb140, which was isolated after treatment of the yeast-like form of the human isolate IVIC Pb9 with the mutagen nitrosoguanidine. The mutant did not form the whitish cotton-like colonies typically found among the parental colonies. Yeast-like cells of the mutant were much smaller than those of the parent and formed chains of different sizes; α-1,3-glucan, which was typically observed in the cell wall of the parental strain, was replaced by amorphous 1,3-mannan in the mutant [67]. San-Blas and Vernet [68] reported that in vitro subculturing of the yeast-like form of IVIC Pb9 led to the disappearance of cell wall α-1,3-glucan, but the addition of fetal calf serum to the growth medium induced the synthesis of α-1,3-glucan. Therefore, the authors suggested that α-1,3-glucan levels in the cell wall of the yeast-like form of IVIC Pb9 are upregulated by external factors [68].

In the yeast-like form of IVIC Pb9, the α-1,3-glucan content in the cell wall was reduced from 45% to 3% after subculture in vitro for several years, but was partially restored (up to 25%) upon growth in vivo [69]. The IVIC Pb140 mutant could not be recovered from experimentally infected animals. The absence of α-1,3-glucan in IVIC Pb140 suggests a relationship between the presence of α-1,3-glucan in the cell wall and pathogenicity of *P.* *brasiliensis* [69]. Using the same approach, San-Blas et al. [70] isolated an adenine-requiring mutant, IVIC Pb141. In the cell wall of this mutant, α-1,3-glucan content was increased and antigenic galactomannan virtually disappeared. The higher virulence of IVIC Pb141 confirmed the relationship between cell wall α-1,3-glucan and the pathogenicity in *P. brasiliensis* [70].

In *H. capsulatum*, cell wall α-1,3-glucan was analyzed by Klimpel and Goldman [65], who isolated spontaneous isogenic avirulent clones from virulent strains. On plates, avirulent colonies (G186AS) had a smooth texture, whereas virulent colonies (G186AR) were rough and convoluted. In liquid medium, the avirulent strains did not aggregate, whereas the parental strains aggregated. In mice, the 50% lethal dose values of the smooth variants were similar to those of an avirulent strain [65]. Growth curves for the rough and smooth variants were similar. No differences in protein profiles were detected in crude cell fractions on one-dimensional polyacrylamide gels or in whole-cell extracts on two-dimensional gels. Electrophoresis of culture supernatants, however, revealed a difference in a released low-molecular-weight peptide that may be related to virulence. The same authors revealed that avirulent *H. capsulatum* strains contained up to 1000-fold less α-1,3-glucan than did their virulent parents, and no α-1,3-glucan could be detected on the surface of the avirulent strain with a mouse monoclonal antibody against α-1,3-glucan [71]. The authors concluded that α-1,3-glucan is an important common virulence determinant.

Due to a similar relationship between virulence and α-1,3-glucan had been described for *P. brasiliensis* and *H. capsulatum,* Hogan and Klein [72] analyzed this relationship in three genetically-related strains of *B. dermatitidis*: the wild-type virulent strain ATCC 26199; mutant strain ATCC 60915 with 10,000-fold lower virulence; and avirulent mutant strain ATCC 60916 [72]. Immunologic quantitation revealed that both mutant strains almost completely lost cell-wall α-1,3-glucan, in contrast to its high concentration in the wild-type strain. These results suggested that there were alterations of yeast surface expression of the WI-1 antigen and recognition and binding of the related strains by human monocyte-derived macrophages [72].

Rappleye et al. [9] obtained direct evidence that α-1,3-glucan is an important contributor to the virulence of *H. capsulatum*. RNAi targeting *AGS1* (which encodes α-1,3-glucan synthase) led to a depletion of cell wall α-1,3-glucan, which attenuated the ability of *H. capsulatum* to kill macrophages and colonize murine lungs, a phenotype that was indistinguishable from that conferred by *AGS1* deletion [9]. Rappleye et al. [10] also revealed that α-1,3-glucan is present in the outermost layer of the *H. capsulatum* cell wall and contributes to pathogenesis by concealing immunostimulatory β-glucans from detection by host phagocytic cells. The production of the proinflammatory cytokine TNFα by phagocytes was suppressed either by the presence of the α-1,3-glucan layer or by RNAi-mediated depletion of the host β-glucan receptor Dectin-1 [10].

*H. capsulatum* is classified into two chemotypes based on cell wall composition [73]. The cell wall of chemotype II contains a layer of α-1,3-glucan, which is essential for virulence [74]. Chemotype I lacks α-1,3-glucan in vitro but is fully virulent in vivo*.* To clarify whether *AGS1* contributes to the virulence of chemotype I, the function of *AGS1* was inhibited by RNAi or by insertional mutagenesis [74]. Cytotoxicity was not impaired by the loss of *AGS1* function: despite the absence of cell wall α-1,3-glucan, chemotype I yeast could avoid detection by Dectin-1. These results indicate that *AGS1* is dispensable for chemotype I virulence, in contrast to chemotype II, in which *AGS1* is essential for the synthesis of cell wall α-1,3-glucan. These results also suggest that the production of a unique chemotype I factor at least partially circumvents the α-1,3-glucan requirement for virulence [74].

In *H. capsulatum* and *P. brasiliensis*, the production of cell wall α-1,3-glucan was found to require the function of the *AMY1* gene product, which was a novel protein with a sequence similarity to that of α-amylases from the GH13_5 subfamily [59,60]. Deletion of *H. capsulatum AMY1* attenuated the pathogen’s ability to kill macrophages and to colonize murine lungs [59]. In an *H. capsulatum amy1* mutant strain complemented with the *P. brasiliensis AMY1* gene [60], the transcription level of *P. brasiliensis* *AMY1* increased during the yeast phase and was correlated with the presence of cell wall α-1,3-glucan. Complemented cells not only synthesized cell wall α-1,3-glucan, but also became virulent. These findings suggest that *P. brasiliensis* Amy1p is involved in cell wall α-1,3-glucan biosynthesis and virulence [60].

The importance of cell wall α-1,3-glucan for virulence was reported also in *Cryptococcus neoformans*. In in this species, cell wall α-1,3-glucan is required for the association of the capsule with the cell [75]. Cells with α-1,3-glucan synthase expression suppressed by RNAi grew slowly and were unable to assemble a capsule, although they generated its polysaccharide components [75]. Reese et al. [76] analyzed *C. neoformans* cells with disrupted α-glucan synthase gene (Δ*ags1*). Transmission electron microscopy and analysis of cell wall composition revealed that these cells lacked cell wall α-1,3-glucan and capsule fibers. Although these cells were able to secrete the capsule material, the capsule was absent on the cell surface. The mutant cell wall was thickened and ragged and the cells had a hypertrophic inner zone. The complete loss of α-1,3-glucan led to a compensatory increase in chitin/chitosan and a redistribution of β-glucan between the cell wall alkali-soluble and insoluble fractions. Although the mutants showed toxicity in the nematode model, the mutants did not show virulence in the mouse model of infection [76].

#### 4.3.2. α-1,3-Glucan Is a Virulence Factor in *Aspergillus fumigatus*

The importance of cell wall α-1,3-glucan for virulence was reported in filamentous fungi, in particular for the most important airborne fungal pathogen *A. fumigatus* (Figure 3D). To analyze the virulence of *A. fumigatus* wild-type and mutant strains and the role of α-1,3-glucan in virulence, Beauvais et al. [29] developed an experimental model of invasive aspergillosis based on mixed infection and quantitative PCR. As mentioned in Section 2.2, *A. fumigatus* has three α-1,3-glucan synthase genes (*AGS1*, *AGS2*, and *AGS3*) [2,29,30], and the deletion of *AGS1* led to a 50% decrease in the level of cell wall α-1,3-glucan, whereas deletion of *AGS2* had no detectable effect on glucan levels. Deletion of either *AGS1* or *AGS2* yielded cells with altered hyphal morphology and reduced conidiation [29]. Immunolocalization revealed that Ags1p was close to the cell wall, whereas Ags2p was intracellular. The pathogenicity of the *AGS1* deletion mutant was similar to that of the wild-type [29].

The strain lacking *AGS3*, in which *AGS1* expression was increased, demonstrated faster and more robust disease development than did the parental strain in a mouse model of aspergillosis [30]. In this strain, the increased conidial melanization, the increased resistance to reactive oxygen species, and the faster germination of conidia led to hypervirulence. Thus, *AGS3* indirectly affects virulence by affecting the response to environmental changes [30].

Henry et al. [15] reported the generation of a triple-mutant strain of *A. fumigatus* that lacked *AGS1*, *AGS2*, and *AGS3*, and had no cell wall α-1,3-glucan, which led to an increase in β-1,3-glucan and chitin levels. This mutant was less pathogenic than the parental strain in a murine aspergillosis model [11]. The growth rate of the triple mutant was similar to that of the parental strain under several stress conditions, such as hypoxia or the presence of reactive oxidants, cationic peptides, or antifungal molecules. However, after phagocytosis by murine alveolar macrophages isolated from the broncho-alveolar lavage, the killing rate of Δ*ags* conidia was significantly higher than that of the parental strain. The presence of an amorphous glycoprotein matrix that covers the rodlet layer (see Section 2.2) was responsible for the reduction of the viability of conidia in vivo and the decrease in virulence [11]. Recently, α-1,3-glucan of *A. fumigatus* has been proposed to induce the maturation of human dendritic cells [77]. Further research is necessary to elucidate the relationships between α-1,3-glucan and the virulence of this fungus.

#### 4.3.3. α-1,3-Glucan Is a Virulence Factor in *Magnaporthe grisea*

*Magnaporthe grisea* is the causal agent of rice blast disease, which leads to enormous economic damage in rice production. Fujikawa et al. [12] demonstrated that α-1,3-glucan of *M. grisea* is a stealth factor that prevents recognition by the host (Figure 3E). They investigated localization of major cell wall polysaccharides during infectious structure development in *M. grisea* by cytological analysis with fluorescent labels. Although α-1,3-glucan accumulated on both germ tubes and appressoria developing on plant surfaces, it was detectable only on appressoria on plastic coverslips. β-1,3-Glucan and chitin became detectable in infectious hyphae only after digestion of α-1,3-glucan with α-1,3-glucanase. The fungal cell wall became more tolerant to chitinase digestion upon accumulation of α-1,3-glucan. These observations suggest that α-1,3-glucan masks β-1,3-glucan and chitin, which are recognized by the plant innate immune system, and protects the fungal cell wall from digestive enzymes produced by plant cells during infection, thus hampering recognition by host cells. The expression of the gene for α-1,3-glucan synthase (*MoAGS1*) and accumulation of α-1,3-glucan in *M. grisea* depends on Mps1p MAP kinase, whereas the expression of genes encoding other cell wall–related enzymes, such as β-1,3-glucan synthase and chitin synthase, is Mps1p-independent [12]. Fujikawa et al. [13] analyzed the function of cell wall α-1,3-glucan using an *M. oryzae* mutant, *ags1*, which lacks *MoAGS1*. This mutant showed normal appressorium formation on glass coverslips (inductive surface) and during development of infectious hyphae in cells of heat-killed rice or onion epidermis. However, most of these appressoria were not melanized and were, thus, destroyed before penetration into the plant, resulting in the loss of pathogenicity in susceptible host plants [13]. This suggests that defense responses of the host plant were activated by exposed β-1,3-glucan and chitin. This suggestion is consistent with the observation that transgenic rice plants expressing *Bacillus circulans* α-1,3-glucanase (*AGL*) rapidly activated defense responses upon infection with wild-type *M. oryzae* [13]. The *AGL*-expressing rice plants showed strong resistance not only to *M. oryzae*, but also to the phylogenetically distant ascomycete *Cochliobolus miyabeanus* and the polyphagous basidiomycete *Rhizoctonia solani* [13]. Treatment with α-1,3-glucanase in vitro caused fragmentation of infectious hyphae in *R. solani*, indicating that α-1,3-glucan is also involved in maintaining the integrity of infectious structures in some fungi. On the basis of these observations, Fujikawa et al. [13] suggested that many fungal plant pathogens use α-1,3-glucan for innate immune evasion and some use it to maintain infectious structures, because α-1,3-glucan is non-degradable in plants.

## 5. Industrial Applications of α-1,3-Glucan Mutants

The dispersibility of the hyphae of α-1,3-glucan-deficient mutants can be used for high-cell-density cultivation, which is effective for high productivity in industrial fermentation. As mentioned above, the hyphae of an *A. nidulans* α-1,3-glucan-deficient strain are completely dispersed in liquid medium [16]. The biomass of this strain was significantly higher than that of the wild-type strain in liquid culture [17]. Filamentous fungi have been used in the fermentation industry because they secrete enzymes and metabolites of high commercial value. Although secretion levels are higher in filamentous fungi than in yeasts and bacteria, their hyphae often clump together and form large pellets, which limits cell density in liquid culture. We were inspired by the phenotype of the *A. nidulans* α-1,3-glucan-deficient strain and started research on its application to high-density culture. First, we constructed a triple deletion (∆*agsA*∆*agsB*∆*agsC*; triple∆) strain of the α-1,3-glucan synthase genes in industrial fungus *A. oryzae* [17]. In liquid culture, this strain formed smaller hyphal pellets and had higher cell dry weight than those of the wild-type. Next, we introduced the cutinase-encoding gene *cutL1* into wild-type *A. oryzae* (wild-type-cutL1) and the tripleΔ mutant (tripleΔ-cutL1). As expected, tripleΔ-cutL1 formed smaller hyphal pellets and had greater biomass and CutL1 productivity than wild-type-cutL1. Smaller hyphal pellets of tripleΔ-cutL1 were more tolerant to hypoxia and hypoxia-induced autolysis than the larger pellets of wild-type-cutL1. Increased CutL1 productivity of tripleΔ-cutL1 was also confirmed under conditions mimicking industrial conditions in which culture media often contain high concentrations of sucrose, glucose, or fructose. The increased CutL1 productivity achieved by tripleΔ-cutL1 might be attributable to a decrease in the number of tripleΔ-cutL1 cells under anaerobic conditions [17].

Fungal β-glucans are recognized by Dectin-1 in the human immune system [78]. Therefore, the availability of α-1,3-glucan-deficient mutants in *A. oryzae* may lead to the development of strains with increased ability to promote immune responses. Thus, isolation of α-1,3-glucan-deficient strains would be expected to lead to the development of high-value-added production of functional foods, including the cells of koji mold, which is used to produce *miso* and *amazake* (traditional Japanese sweet drinks made from fermented rice). Since the food use of genetically-modified organisms is not permitted in Japan, to isolate α-1,3-glucan-deficient strains of *A. oryzae*, we used chemical mutagenesis of conidia and a screening method based on the increased sensitivity of these stains to CR [79]. We isolated several candidate mutants with increased sensitivity to CR and/or enzymatic hydrolysis. Cytokine production was increased in dendritic cells co-incubated with the germinated conidia of these mutants [79].

Development of bacterial α-1,3-glucan as a high-performance material is also underway [18]. Unnatural linear α-1,3-glucan with no branches was synthesized by in vitro polymerization with recombinant GtfJ, a glucosyltransferase from *Streptococcus salivarius* [18]. Acetate and propionate esters of this α-1,3-glucan (crystalline α-1,3-glucan esters) were then synthesized and characterized [18]. The melting temperature of α-1,3-glucan acetate was higher than that of commercially-available thermoplastics, such as polyethylene terephthalate and Nylon 6 [18]. Thus, the discovery of unbranched crystalline α-1,3-glucan esters with high thermal stability and melting temperature opens the gate for further studies on their application as thermoplastic materials [18]. Although α-1,3-glucan of the fungal cell wall contains a small amount of the primer (α-1,4-linked oligosaccharide) and has 1,3/1,6 branching, the fungal α-1,3-glucan showing various biological functions as described might also be available as a novel biological material.

## 6. Conclusions and Perspectives

In the past decade, studies of cell wall architecture and biogenesis in filamentous fungi have advanced greatly owing to the availability of genome sequence information and the development of genome-wide analysis tools. In this review, we have described our current understanding of the biological functions and biosynthesis of cell wall α-1,3-glucan. Biosynthesis of linear α-1,3-linked glucan, which is the main chain of fungal α-glucan, is largely similar in filamentous fungi and *S. pombe*, whereas some differences in biosynthetic mechanisms are observed among fungi. For instance, the SYN domain of *S. pombe* Ags1p may be capable of synthesizing oligo α-1,4-glucan, which is a primer for α-1,3-glucan synthesis, but the oligo α-1,4-glucan primer may be synthesized by the α-amylase AmyG in *A. nidulans*. Filamentous fungi contribute greatly to global biomass circulation by decomposing various substrates. Fungal hyphae invade substrates during fermentation, which mimics infection of plants and animals by pathogenic fungi. Therefore, further studies of cell wall α-1,3-glucan might accelerate our understanding of its biological roles in both fermentation and infection by filamentous fungi.

## Figures and Tables

**Table 1:** Genes related to α-1,3-glucan synthesis and degradation in *Aspergillus* species.

*A. nidulans*		*A. fumigatus*		*A. oryzae*		*A. niger*	
Gene	Phenotypes	Gene	Phenotypes	Gene	Phenotypes	Gene	Phenotypes
	**α-1,3-Glucan synthases**						
*agsA*	Expressed at a very low level [16] High expression level in Δ*mpkA* strain [32]	*AGS2*	No change in α-1,3-glucan content in Δ*ags2* strain [29]	*agsA*	Expressed at a very low level [36]	*agsD*	Not reported
*agsB*	Main α-1,3-glucan synthase [16] Hyphal aggregation [16] Upregulated by micafungin treatment [32]	*AGS1*	50% reduction in α-1,3-glucan content in Δ*ags1* strain [29]	*agsB*	Major α-1,3-glucan synthase [36]	*agsE*	Upregulated by CFW treatment [31]
		*AGS3*	Hypervirulence in Δ*ags3* strain [30]	*agsC*	Not directly involved in α-1,3-glucan synthesis [36]	*agsA*	Upregulated by CFW treatment [31]
						*agsB*	Not reported
						*agsC*	Downregulated by CFW treatment [31]
	**Intracellular α-amylases**						
*amyG*	Important for α-1,3-glucan synthesis [33,37]	1	Not reported	2	Not reported	*amyD*	Starch hydrolysis [38]
						*1*	Not reported
	**GPI-anchored α-amylases**						
*amyD*	Degradation of α-1,3-glucan [33,37]	3	Not reported	2	Not reported	*agtA*	α-1,4-Glucano-transferase [34]
						*agtB*	α-1,4-Glucano-transferase [34]
						*agtC*	Not reported
	**α-1,3-Glucanases**						
*mutA*	Hülle cell localization [24] Hydrolysis of α-1,3-glucan [37]		Not annotated		Not annotated	*agnB*	α-1,3-Glucan hydrolysis [39]
*agnB*	Hydrolysis of α-1,3-glucan [37]					*agnE*	Not reported
*agnE*	No α-1,3-glucan hydrolysis [37]						
2	Not reported						

CFW, calcofluor white. Numbers in the “Gene” column indicate additional uncharacterized genes.
